# Supplementary material for: Taller height and risk of coronary heart disease and cancer: A within-sibship Mendelian randomization study
Source: eLife. 2022 Mar 18;11:e72984. doi: 10.7554/eLife.72984 (PMC8947759; doi:10.7554/eLife.72984)
Supplement: Supplementary file 1. — (a) contains the different variables and codes used to define coronary heart disease, all cancers, and subset of cancer disease outcomes in UK Biobank. (b) contains population and within-sibship associations between measured height and the eight different outcomes. Presented estimates are from UK Biobank, HUNT, and the combined fixed-effects meta-analysis. The heterogeneity p-value refers to the difference between the UK Biobank and HUNT estimates. (c) contains population and within-sibship associations between the height polygenic score (PGS) and the eight different outcomes. Estimates are presented from UK Biobank and HUNT. (d) contains population and within-sibship associations between three height-related measures (leg length, trunk length, and leg/trunk ratio) and cancer and coronary heart disease from UK Biobank. Height associations are presented for comparison. (e) contains population and within-sibship phenotypic and Mendelian randomization estimates for height on a set of cancer subtypes with limited prior evidence for association with height. Estimates for the ‘all cancers’ outcome are included for comparison. This analysis was conducted in UK Biobank only. [file elife-72984-supp1.docx]

**Supplementary File 1A** Definitions of coronary heart disease and cancer in UK Biobank

| **Phenotype** | **Data source (codes)** |
| --- | --- |
| Coronary heart disease | Hospital Episode Statistics (ICD10: I21-I25, Z95)  Mortality registry (ICD10: I21-I25, Z95)  Hospital Procedures (OPCS: K40-K46, K471, K49, K50, K75) |
| Cancer (all cancers) | Hospital Episode Statistics (ICD10: Any C code)  Cancer registry (ICD10: Any C code)  Mortality registry (ICD10: Any C code) |
| Subset of cancers* | Hospital Episode Statistics (ICD10: C34, C0, C15-16, C25, C67, C90)  Cancer registry (ICD10: C34, C0, C15-16, C25, C67, C90)  Mortality registry (ICD10: C34, C0, C15-16, C25, C67, C90) |

* Cancers with limited evidence for associations with height (lung, oropharyngeal, stomach, oesophageal, pancreatic, bladder and multiple myeloma)

Supplementary File 1A contains the different variables and codes used to define coronary heart disease, all cancers and subset of cancers disease outcomes in UK Biobank.

**Supplementary File 1B** Phenotypic results: Change in outcome (SD units), per 1 S.D. increase in height.

| **Outcome** | **Model** | **UK Biobank** | **HUNT** | **Combined** | **Study heterogeneity**  **P-value** |
| --- | --- | --- | --- | --- | --- |
| Systolic blood pressure | Population | -0.066 (-0.076, -0.056) | -0.040 (-0.051, -0.029) | -0.054 (-0.061, -0.046) | < 0.001 |
|  | Within-sibship | -0.042 (-0.061, -0.024) | -0.006 (-0.022, 0.010) | -0.021 (-0.033, -0.009) | 0.004 |
| HDL cholesterol | Population | 0.013 (0.002, 0.024) | -0.008 (-0.019, 0.003) | 0.003 (-0.005, 0.011) | 0.010 |
|  | Within-sibship | -0.030 (-0.048, -0.012) | -0.031 (-0.046, -0.015) | -0.030 (-0.042, -0.019) | 0.95 |
| LDL cholesterol | Population | -0.018 (-0.029, -0.008) | -0.033 (-0.044, -0.022) | -0.025 (-0.033, -0.018) | 0.059 |
|  | Within-sibship | -0.022 (-0.040, -0.003) | -0.030 (-0.046, -0.015) | -0.027 (-0.039, -0.015) | 0.47 |
| Triglycerides | Population | -0.044 (-0.054, -0.034) | -0.014 (-0.026 -0.003) | -0.030 (-0.038, -0.023) | < 0.001 |
|  | Within-sibship | -0.010 (-0.028, 0.008) | 0.005 (-0.012, 0.022) | -0.002 (-0.015, 0.010) | 0.24 |
| Glucose | Population | -0.018 (-0.029, -0.007) | N/A | -0.018 (-0.029, -0.007) | N/A |
|  | Within-sibship | -0.008 (-0.029, 0.011) | N/A | -0.008 (-0.029, 0.011) | N/A |
| IGF-1 | Population | 0.070 (0.060, 0.081) | N/A | 0.070 (0.060, 0.081) | N/A |
|  | Within-sibship | 0.050 (0.032, 0.068) | N/A | 0.050 (0.032, 0.068) | N/A |
| Cancer (OR) | Population | 1.10 (1.07, 1.13) | 1.00 (0.96, 1.04) | 1.05 (1.02, 1.07) | 0.006 |
|  | Within-sibship | 1.09 (1.04 1.15)) | 1.04 (0.96, 1.11) | 1.05 (1.01, 1.09) | 0.71 |
| Coronary heart disease (OR) | Population | 0.83 (0.80, 0.86) | 0.92 (0.89, 0.94) | 0.89 (0.87, 0.91) | < 0.001 |
|  | Within-sibship | 0.86 (0.80, 0.92) | 0.98 (0.94, 1.03) | 0.95 (0.92, 0.99) | 0.001 |

Supplementary File 1B contains population and within-sibship associations between measured height and the 8 different outcomes. Presented estimates are from UK Biobank, HUNT and the combined fixed-effects meta-analysis. The heterogeneity P-value refers to the difference between the UK Biobank and HUNT estimates.

**Supplementary File 1C** Change in outcome (SD units), per 1 S.D. increase in height PGS.

| **Outcome** | **UK Biobank** | |  | **HUNT** | |
| --- | --- | --- | --- | --- | --- |
|  | **Population** | **Within-sibship** |  | **Population** | **Within-sibship** |
| Height | 0.361 (0.350, 0.371) | 0.311 (0.298, 0.323) |  | 0.335 (0.324, 0.346) | 0.334 (0.321, 0.346) |
| Systolic blood pressure | -0.016 (-0.027, -0.005) | -0.024 (-0.042, -0.005) |  | -0.008 (-0.019, 0.003) | 0.003 (-0.013, 0.020) |
| HDL cholesterol | -0.014 (-0.025, -0.003) | -0.012 (-0.030, 0.006) |  | -0.003 (-0.14, 0.008) | 0.000 (-0.015, 0.016) |
| LDL cholesterol | -0.024 (-0.034, -0.013) | -0.026 (-0.044, -0.008) |  | -0.022 (-0.033, -0.011) | -0.005 (-0.020, 0.011) |
| Triglycerides | 0.004 (-0.006, 0.015) | 0.007 (-0.010, 0.025) |  | -0.002 (-0.13, 0.009) | 0.011 (-0.006, 0.027) |
| Glucose | 0.012 (0.002, 0.022) | 0.008 (-0.011, 0.028) |  | NA | NA |
| IGF-1 | -0.002 (-0.012, 0.009) | -0.016 (-0.034, 0.001) |  | NA | NA |
| Cancer (OR) | 1.04 (1.02 1.07) | 1.06 (1.01, 1.12) |  | 1.00 (0.96, 1.04) | 1.04 (0.96, 1.12) |
| Coronary heart disease (OR) | 0.98 (0.94, 1.01) | 0.94 (0.87, 1.01) |  | 0.96 (0.93, 0.98) | 0.96 (0.92, 1.00) |

Supplementary File 1C contains population and within-sibship associations between the height PGS and the 8 different outcomes. Estimates are presented from UK Biobank and HUNT.

**Supplementary File 1D** Associations of measured leg and trunk length with risk of cancer and coronary heart disease in UK Biobank

| **Outcome** | **Model** | **OR of outcome per SD increase (95% C.I.)** | | | |
| --- | --- | --- | --- | --- | --- |
|  |  | **Height* (REF)** | **Leg length** | **Trunk length** | **Leg/trunk ratio** |
| Cancer | Population | 1.10 (1.07, 1.13) | 1.09 (1.06, 1.12) | 1.07 (1.04, 1.10) | 1.04 (1.01, 1.06) |
|  | Within-sibship | 1.09 (1.04, 1.15) | 1.07 (1.02, 1.12) | 1.05 (1.01, 1.10) | 1.02 (0.97, 1.06) |
| Coronary heart disease | Population | 0.83 (0.80, 0.86) | 0.84 (0.81, 0.88) | 0.89 (0.86, 0.92) | 0.93 (0.90, 0.97) |
|  | Within-sibship | 0.86 (0.80, 0.92) | 0.89 (0.84, 0.96) | 0.92 (0.86, 0.98) | 0.97 (0.92, 1.04) |

Supplementary File 1D contains population and within-sibship associations between three height-related measures (leg length, trunk length and leg/trunk ratio) and cancer and coronary heart disease from UK Biobank . Height associations are presented for comparison.

**Supplementary File 1E** Height and cancer subtypes with limited evidence for associations with height in UK Biobank

| **Analysis** | **Model** | **OR of cancer per SD increase in height (95% C.I.)** | |
| --- | --- | --- | --- |
|  |  | **All cancers (REF: 9,146 cases)** | **Subset of cancers* in UK Biobank (909 cases)** |
| Phenotypic | Population | 1.05 (1.02, 1.07) | 0.99 (0.92, 1.06) |
|  | Within-sibship | 1.05 (1.01, 1.09) | 1.01 (0.88, 1.15) |
| Mendelian randomization | Population | 1.09 (1.02, 1.16) | 1.11 (0.93, 1.35) |
|  | Within-sibship | 1.18 (1.03, 1.34) | 1.23 (0.81, 1.86) |

* Cancers with limited evidence for associations with height (lung, oropharyngeal, stomach, oesophageal, pancreatic, bladder and multiple myeloma)

Supplementary File 1E contains population and within-sibship phenotypic and Mendelian randomization estimates for height on a set of cancer subtypes with limited prior evidence for association with height. Estimates for the “all cancers” outcome were included for comparison. This analysis was conducted in UK Biobank only.
